# Training and deployment of lay refugee/internally displaced persons to provide basic health services in camps: a systematic review

**DOI:** 10.3402/gha.v7.23902

**Published:** 2014-10-01

**Authors:** John E. Ehiri, Jayleen K.L. Gunn, Katherine E. Center, Ying Li, Mae Rouhani, Echezona E. Ezeanolue

**Affiliations:** 1 Division of Health Promotion Sciences/Global Health Institute, Mel & Enid Zuckerman College of Public Health, University of Arizona, Tucson, AZ, USA; 2 Division of Epidemiology and Biostatistics, Mel & Enid Zuckerman College of Public Health, University of Arizona, Tucson, AZ, USA; 3 Department of Obstetrics and Gynecology, University of Arizona, Tucson, AZ, USA; 4 Department of Social Medicine & Health Service Management, Third Military Medical University, Chongqing, China; 5 Department of Pediatrics, University of Nevada School of Medicine, Las Vegas, NV, USA

**Keywords:** maternal health, reproductive health, refugees, internally displaced persons, child health

## Abstract

**Background:**

Training of lay refugees/internally displaced persons (IDPs) and deploying them to provide basic health services to other women, children, and families in camps is perceived to be associated with public health benefits. However, there is limited evidence to support this hypothesis.

**Objectives:**

To assess the effects of interventions to train and deploy lay refugees and/or IDPs for the provision of basic health service to other women, children, and families in camps.

**Methods:**

PubMed, Science and Social Science Citation Indices, PsycINFO, EMBASE, POPLINE, CINAHL, and reference lists of relevant articles were searched (from inception to June 30, 2014) with the aim of identifying studies that reported the effects of interventions that trained and deployed lay refugees and/or IDPs for the provision of basic health service to other women, children, and families in camps. Two investigators independently reviewed all titles and abstracts to identify potentially relevant articles. Discrepancies were resolved by repeated review, discussion, and consensus. Study quality assessment was undertaken using standard protocols.

**Results:**

Ten studies (five cross-sectional, four pre-post, and one post-test only) conducted in Africa (Guinea and Tanzania), Central America (Belize), and Asia (Myanmar) were included. The studies demonstrated some positive impact on population health associated with training and deployment of trained lay refugees/IDPs as health workers in camps. Reported effects included increased service coverage, increased knowledge about disease symptoms and prevention, increased adoption of improved treatment seeking and protective behaviors, increased uptake of services, and improved access to reproductive health information. One study, which assessed the effect of peer refugee health education on sexual and reproductive health, did not demonstrate a marked reduction in unintended pregnancies among refugee/IDP women.

**Conclusion:**

Although available evidence suggests a positive impact of training and deployment of lay refugees/IDPs as health workers in camps, existing body of evidence is weak, and calls for a re-examination of current practices. Interventions that promote training and deployment of lay refugees/IDPs as health workers in camps should include strong evaluation components in order to facilitate assessment of effects on population health.

Although the value of using local community health workers in the provision of basic health services is widely acknowledged, many health and development agencies working in camps for refugees and internally displaced persons (IDPs) continue to rely on expatriate health workers. Often, this dependence raises concerns about the long-term sustainability, cost-effectiveness, and cultural appropriateness of programs and services. The focus of this review is to summarize and critically appraise evidence regarding both the capacity of refugee and/or internally displaced women residing in camps to provide health services to other women and children in these camps and the effectiveness of such services. Anecdotal evidence suggests that with adequate training, proper supervision, and support, refugee and/or internally displaced women are able to provide basic health services to other women, children and families in camps while avoiding the challenge of ensuring cultural appropriateness and sustainability that are often associated with transient expatriate personnel.

A refugee is defined as someone who has fled his or her country of nationality to find protection from war or from persecution based on race, religion, nationality, membership of a specific political party and/or political opinion (1, 2). ‘Unlike refugees, IDPs have not crossed an international border to find sanctuary, but have remained inside their home countries. As such, IDPs are legally under the protection of their own government, even if that government was the cause of their flight’ (2, p. 1). Also included in the definition of IDPs are civilians who are made homeless by natural disasters (3). At the end of 2011, the United Nations High Commission for Refugees (UNHCR) reported that they were providing services for an estimated 26.4 million people worldwide who have been displaced because of conflict or persecution (3). Of this number, an estimated 10.4 million were refugees and 15.6 million were IDPs (3). In addition, UNCHR estimates that nearly half of the population of refugees and IDPs were females (3). Globally, sub-Saharan Africa is disproportionately affected by conflicts and emergencies that have resulted in large numbers of refugees and IDPs (34% of the global total) (4). Political, religious, and ethnic disputes are significant factors contributing to these high numbers. For example, the genocide in Rwanda resulted in 1.7 million refugees; the conflicts in Liberia 750,000, and the Somalia conflict, 450,000 (5).

As the world continues to experience increases in conflicts and emergencies, health workers and international health agencies are confronted with the challenge of protecting the health of displaced populations. Women, infants, and children are often severely affected, not by the direct effects of weapons, but by displacement, gender-based violence, preventable illness, malnutrition, and inadequate sexual and reproductive health services (6). Women and children are at increased risk for poor health when community resources and networks that serve as their safety nets are disrupted. Sexual violence and abuse are also of particular concern for women and girls (7–10).

## Why it is important to do this review

As available evidence shows, major health challenges that often confront refugee and internally displaced women, children, adolescents and families include: 1) limited access to health services, 2) increased predisposition to sexual and reproductive health risks, 3) under-nutrition, and 4) chronic disruption of services. Although several international initiatives have sought to unite aid agencies that respond to the needs of refugees and IDPs (11), attempts to empower displaced women in camps to take control over, and improve their own health have been limited. With the escalation of conflicts and emergencies globally, gaps in access to essential health services for women and children in camps continue to occur (12–14). With the flight of local health workers, the burden of health services typically rests on just a few foreign aid workers, severely limiting service coverage (15, 16). This problem calls for a paradigm shift in efforts to address the maternal and child-health needs of refugees and IDPs in camps using available local human resources. Over the years, a number of intervention studies have been conducted in which refugee and/or internally displaced women residing in camps were provided with targeted training in specific basic health services related to maternal, newborn, and child-health services to other women and families in their camps, and later deployed to apply the resultant knowledge and skills. Anecdotal evidence suggests that such training and deployment have public health benefits but few reviews have articulated this evidence. This review therefore, is an effort to address this gap in knowledge.

## Methods

### Search strategy and selection criteria

The following electronic databases were searched from inception to June 30, 2014: PsycINFO, PubMed, Web of Science, CINAHL, Sociological Abstracts, Embase, and Internet (Google and Google Scholar). The search was not restricted by publication status or language. The following search terms were first created for a search in PubMed, and later adapted for the other databases: (‘Community Health Workers’ OR ‘Health Personnel’ OR ‘Volunteers’ OR ‘Health Services’ OR ‘Delivery of Health Care’ OR volunteer OR ‘health worker’ OR ‘health auxiliary’ OR ‘relief work’ OR ‘relief worker’ OR ‘health care delivery’ OR peer); (‘Refugees’ [Mesh] OR ‘refugee camps’ OR ‘internally displaced’); #1 AND #2. We hand-searched reference lists of identified articles, and contacted bilateral agencies and non-governmental organizations (NGOs) whose programs may include interventions for refugees and/or IDPs with a request for information on previous and ongoing studies. Three reviewers (JE, JG, and YL) coordinated the literature searches.

### Inclusion/exclusion criteria

1) Types of study: We attempted to identify studies that assessed the capacity of lay women who were refugees or internally displaced, to provide basic health services to maternal and child populations (women, children, and adolescents) in camps for refugees and/or IDPs and the outcomes of such services. We excluded repeated reports, books, studies that provided general discussion about training of non-refugee community health workers, and descriptive papers on refugees and IDPs in camp settings. Studies that assessed the effectiveness of services provided by trained local or expatriate health workers such nurses, doctors, and other skilled medical and allied health professionals. Given the ethical and logistical challenges of conducting randomized controlled trials under camp settings and the dearth of empirical evidence on the subject, we sought to include a wide variety of studies in order to capture as much available data as possible. Thus, we included cohort studies, cross-sectional studies, controlled pre-and-post studies, controlled post-test studies, and pre-posttest studies that included an evaluation component. 2) Study population: Females aged 15 years and over, who were residing in camps for refuges/IDPs, and who were involved in providing basic health services to women, children adolescents and families in camps settings or who had received training to provide such services. 3) Outcome measures: a) Changes in health-related knowledge, attitudes, and practices of refugee or internally displaced lay women as a result of training aimed at equipping them to provide basic health services to women, children, adolescents and families in camps; b) changes in health outcomes of women, children, adolescents and families in camps as a result of services provided by trained lay women who were refugees or internally displaced (e.g. changed sexual risk behaviors; fewer unintended pregnancies; increased immunization uptake/coverage; increased uptake of ante-natal and post-natal care and nutrition services; reduced maternal and/or child under-nutrition; increased uptake and use of insecticide-treated bed nets; less frequent malaria cases among women, children, and adolescents; and fewer diarrhea episodes). Two reviewers (JE and JG) applied the inclusion and exclusion criteria to the identified studies.

## Study selection

Two reviewers (JE and KC) independently screened the titles and abstracts of identified studies to assess their eligibility for inclusion in the review. Where uncertainties regarding eligibility of studies occurred, all reviewers participated in the decision about inclusion.

## Data extraction

Data from eligible studies were independently abstracted by two reviewers (JE and JG). Differences were resolved by consensus among all reviewers. Studies were stratified by design (cohort, cross-sectional studies, and case studies). For cohort studies, the number of subjects in the cohort and the number of incident cases of health outcomes of interest in the exposed and non-exposed refugee and internally displaced women, children, adolescents, and families were extracted. For cross-sectional studies, data on the number of persons in the study groups and number of persons exposed/unexposed to health outcomes of interest were extracted from the comparison groups. We also extracted data on sample size, ages of individuals in the study, and data collection methods.

## Study quality assessment

We assessed the quality of cohort studies using the Newcastle–Ottawa Scale (17). In addition, we assessed the representativeness of the exposed cohort in the study setting; the selection of non-exposed cohorts; the ascertainment of exposure; the demonstration that outcome of interest was not present at start of study; the comparability of cohorts on the basis of design and analyses; the outcomes assessments; and the adequacy of follow-up (17). For cross-sectional studies, we used the guidelines for critical appraisal, developed by the National Collaborating Center for Environmental Health (18). We also assessed the representativeness of the study participants; methods for ascertaining exposure; comparability of exposure groups (including unexposed) in terms of age, sex, socioeconomic status, non-response bias, health outcomes, determination, and validation of outcomes; internal validity; and assessment and addressing of confounding factors. Two reviewers (JE and JG) assessed study quality and reached a consensus for each included study.

## Data analysis

We did not conduct statistical meta-analysis given that very few studies qualified for inclusion in the review. More importantly, there was marked heterogeneity in the design and methodology of the included studies, and most did not provide appropriate statistical data to permit meta-analysis or tests of heterogeneity. Thus, we conducted a systematic review by summarizing, comparing, and contrasting the extracted data. The following section presents the results of the systematic review of the 10 eligible studies.

## Results

### Description of included studies

**Fig. 1:**
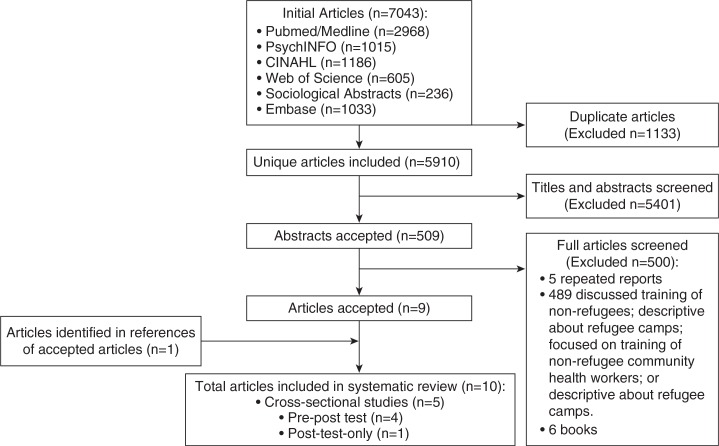
Literature search outputs.

As shown in Figure 1, we included 10 eligible studies (12, 18–27) (five cross-sectional, four pre-post, and one post-test only) conducted in Africa (Guinea and Tanzania) (12, 18–22), Central America (Belize) (23), and Asia (Myanmar) (24–27). These studies were summarized in a systematic review (see Table 1). None of the bilateral agencies and NGOs contacted provided information on evaluated studies of interventions that involved the use of refugees and/or IDPs in providing maternal and child health services in camps. Overall, the literature sources did not yield studies that qualified for inclusion in the review. A detailed discussion of the characteristics of each of the included studies is presented below.

**Table 1:** Characteristics and critical appraisal of included studies

Author (year)	Country	Study design	Objectives	Study population	Intervention and follow-up	Outcomes measured	Key study results	Quality assessment
Chen et al. (2008) (12)	Guinea	Cross-sectional	A) To assess sexual health needs, knowledge and practices among refugees; B) to assess the potential impact of their work, in terms of increased STI knowledge and more appropriate STI-related behavior in clients.	Reproductive-age Liberian and Sierra Leonean men (445) and women (444) from an estimated population of 250,000 refugees living in 48 camps across the Forest Region of Guinea.	RHG recruited refugee nurses and midwives to provide reproductive and sexual health services for refugees in the Forest Region of Guinea, and trained refugee women as lay health workers.	Sexual health needs, knowledge and practices among refugees, e.g. prevalence of reported STI symptoms, knowledge about symptoms and prevention of STIs, treatment seeking and protective behavior adopted by those experiencing STI symptoms, and the potential impact of RHG's work in terms of increased STI knowledge and more appropriate STI-related behaviors.	Respondents citing RHG facilitators as their information source were more likely to respond correctly about STIs; RHG facilitators were more frequently cited than non-healthcare information sources in men who correctly named the key STI symptoms, and in men and women who correctly identified effective STI protection methods.	The analysis did not separate impact of interventions delivered by refugee nurses and midwives from those delivered by trained refugee women who served as lay health workers; cross-sectional studies measure exposure and health outcomes simultaneously. Thus, it is difficult to determine the direction of the observed associations; the study did not measure the relationship between degree of exposure to RHG interventions and health outcomes. Study findings were based on self-reports with high potential for social desirability.
Cropley (2004) (23)	Belize	Post-test only	To assess the effect of health education intervention on child malaria treatment-seeking practices among rural refugee mothers.	Mothers of children aged 6 months to 5 years who resided in eight rural refugee communities.	In four of the eight villages, refugee health workers were trained to provide health education using local beliefs, terminology and disease concepts through one-to-one discussions, informal group meetings and material dissemination.	Changes in knowledge, attitudes, and child fever and malaria treatment-seeking behaviors.	Health education interventions – interpersonal communication in particular – appeared to have a positive effect on fever and malaria beliefs, and on positive treatment-seeking behaviors.	High potential for social desirability; used a post-test only design, with high potential for inadequate comparability of intervention and control communities at baseline. There was potential for contamination of the control communities.
Howard et al. (2008) (19)	Guinea	Cross-sectional	To assess the effect of peer refugee health education on maternal health knowledge, attitudes and behaviors and maternal health outcomes.	Reproductive-age Liberian and Sierra Leonean men (445) and women (444) from an estimated population of 250,000 refugees living in 48 camps across the Forest Region of Guinea.	RHG recruited refugee nurses and midwives and trained refugee women as lay health workers to provide sexual and reproductive and sexual health education.	Effect of peer refugee health education and reproductive service delivery on access to reproductive health information, approval of family planning services, use of contraceptive, perceived service quality, service, risk of unintended pregnancy.	RHG facilitators were the primary source of reproductive health information for all respondents. Contraceptive use in the camps served by RHG was much higher than typical for either refugees’ country of origin or the host country, but the risk of unwanted pregnancy remained considerable.	The analysis did not separate impact of interventions delivered by refugee nurses and midwives from those delivered by trained refugee women who served as lay health workers; cross-sectional studies measure exposure and health outcomes simultaneously. Thus, it is difficult to determine the direction of the observed associations; the study did not measure the relationship between degree of exposure to RHG interventions and health outcomes. Study findings were based on self-reports with high potential for social desirability.
Howard et al. (2011) (20)	Guinea	Cross-sectional	To assess maternal health outcomes in relation to refugee-led health education, formal education, age and parity.	444 reproductive-age Liberian and Sierra Leonean women in 48 camps across the Forest Region of Guinea.	RHG recruited refugee nurses and midwives and trained refugee women as lay health workers to provide sexual and reproductive and sexual health education.	Effect of peer refugee led health education on knowledge of danger signs of complications during pregnancy, knowledge of the importance of skilled attendant at birth, use of family planning, sexual health services, and use of ante-natal care services.	Most respondents said pregnant women should attend antenatal care and knew the importance of tetanus vaccination. Most recognized maternal danger signs and recommended facility attendance for these. Higher odds of facility delivery were found for those exposed to RHG health education. No significant differences were found in knowledge or attitudes.	The analysis did not separate impact of interventions delivered by refugee nurses and midwives from those delivered by trained refugee women who served as lay health workers; cross-sectional studies measure exposure and health outcomes simultaneously. Thus, it is difficult to determine the direction of the observed associations; the study did not measure the relationship between degree of exposure to RHG interventions and health outcomes. Study findings were based on self-reports with high potential for social desirability.
Lee et al. (2009) (26)	Myanmar	Pre- and post-test study	To assess the impact of training and deploying internally displaced villagers on expansion of malaria control interventions among IDPs.	Internally displaced Myanmar villagers.	Staff from the local health department trained internally displaced villagers who lived in the malaria program's target communities and were familiar with other village members. These village health workers were trained in malaria diagnosis and treatment, and vector control and education.	Internally displaced villagers performed malaria diagnosis, treatment, vector control and malaria education in conflict areas.	The intervention demonstrated that internally displaced villagers were able to deliver essential malaria control interventions in areas of active conflict in eastern Burma. Program expanded from 3,000 internally displaced villagers to 40,000 in 5 years.	Although the study demonstrated the ability of trained displaced villages to contribute to significant expansion of malaria treatment and prevention services, evidence of quality or outcomes of services provided by the trained villagers was not provided.
Minden (1997) (27)	Thai-Myanmar Border	Pre- and post-test study	To train refugee community health workers and traditional birth attendants to provide basic reproductive health services, and to diagnose and treat common illnesses using drugs.	Scattered refugee camps in remote areas along the Thai-Myanmar border.	Young camp members (with formal education from grade 4 to grade 10) were trained to diagnose and treat common illnesses. A small subset of the group received 6–18 month medical training. Female community health workers were also selected to receive special training in maternal health and to become midwives. Traditional birth attendants were also trained.	Qualitative outcomes related to complications surrounding pregnancy were discussed.	Trained refugees were able to diagnose and treat common illnesses using drugs, injections and intravenous infusions according to Medecins Sans Frontieres guidelines. They prevented problems, recognized illness early and provided treatment before complications escalated to emergencies. They were able to foresee an emergency and to stabilize the mother and/or baby while they found transportation to a hospital.	Weak design. A more complete evaluation of the quality and health outcomes associated with services provided by the trained refugee health workers is needed to strengthen evidence of health effects of their services.
Mullany et al. (2010) (24)	Myanmar	Pre- and post-test study	To examine the feasibility of a network of community-based providers for delivery of maternal health interventions.	Internally displaced Myanmar women of reproductive age.	In target communities, local health workers and traditional birth attendants were trained in basic emergency obstetric care, blood transfusion, antenatal care and family planning.	Survey to assess effect of the intervention on access to antenatal and postnatal care, presence of skilled attendant at birth, and use of family planning services.	Use of modern methods of birth control increased and births attended by those trained to deliver elements of emergency care increased almost 10-fold.	Weak design. A more complete evaluation of the quality and health outcomes associated with services provided by the trained refugee health workers is needed to strengthen evidence of health effects of their services.
Rijken et al. (2009) (25)	Thai-Myanmar Border	Pre- and post-test study	To assess intra-observer and inter-observer agreement of fetal biometry by locally trained refugee health workers in a refugee camp.	349 pregnant women and local Myanmar health workers.	A 3-month course of practical and theoretical training in obstetric ultrasound imaging based on World Health Organization guidelines and British Medical Ultrasound Society recommendations.	Intra- and inter-observer agreement of fetal biometry measured by trained displaced health workers and those of the expatriate doctor.	Measurements made by local health workers during obstetric ultrasound imaging showed high levels of agreement with those of the doctor.	Weak design. A more complete evaluation of the quality and health outcomes associated with services provided by the trained refugee health workers is needed to strengthen evidence of health effects of their services.
Tanaka et al. (2004) (22)	Tanzania	Cross-sectional	To assess the impact of refugee participation in camp health services provision.	576 refugees, 48 refugee health workers, 17 red cross volunteers, Congolese refugee community members, refugee health workers, and Tanzanian Red Cross staff.	Refugee health workers identified health needs, made health decisions and assumed the responsibility to meet these needs in order to strengthen the refugee community and to improve their health.	Personal profiles of refugee health workers, health status, social support, and knowledge of refugee health workers.	Refugee health workers experienced increased self-confidence and promoted health education. Refugees who did not know a refugee health worker had less positive health seeking behaviors than those who knew a refugee health worker.	The refugees were not formally trained, but instead, used their own initiatives. Being a cross-sectional study, it was not possible to establish causal relationships between the number of refugee HIT members known by refugees and health outcomes.
Woodward et al. (2011) (21)	Guinea	Cross-sectional	To assess whether exposure to peer refugee health education was associated with improved HIV knowledge, attitudes, or practices among refugees.	889 reproductive-age men and women in 23 camps in the Forest Region of Guinea.	RHG recruited refugee nurses and midwives to provide reproductive and sexual health services, and trained refugee women as lay health workers.	Effect of peer-refugee education on HIV knowledge, attitudes, and safe-sex practices.	Refugee-led health education was most strongly associated with reported HIV-avoidant behavior change. Refugee women were more likely to report HIV risk and less likely to report making behavioral changes. Of those exposed to refugee-led education, women had greater odds than men of reporting HIV-avoidant changes.	The analysis did not separate impact of interventions delivered by refugee nurses and midwives from those delivered by trained refugee women who served as lay health workers; cross-sectional studies measure exposure and health outcomes simultaneously. Thus, it is difficult to determine the direction of the observed. associations; the study did not measure the relationship between degree of exposure to RHG interventions and health outcomes. Study findings were based on self-reports with high potential for social desirability.

**Chen et al. [Guinea]** (12). This study-conducted by the Reproductive Health Group (RHG) across the Forest Region of Guinea-used refugee nurses and midwives, and trained lay refugee women as facilitators who provided sexual and reproductive health education to reproductive-aged Liberian and Sierra Leonean refugees living in 48 camps. Through a multistage stratified cluster sampling, 445 men and 444 women were surveyed to assess whether receiving sexual and reproductive health information from lay health workers, trained nurses/midwives, or friends was associated with better knowledge of sexually transmitted infections (STIs) and better health seeking behaviors related to sexual and reproductive health. Overall, participants reported a high prevalence of STIs in the last 12 months (30% women; 24% men). A marked gap in sexual health knowledge was also reported, as only 25% correctly named key symptoms of STIs. Respondents who cited either the refugee nurses/midwives and lay health facilitators as their information source of sexual and reproductive health information demonstrated greater knowledge of STIs. Refugee nurses/midwives or lay health facilitators were more frequently cited than other sources as the suppliers of sexual and reproductive health information by men who correctly named key STI symptoms and by men and women who correctly identified effective STI protection methods.

**Cropley [Belize]** (23). This study used a controlled post-test community-based study to assess the effects of health education intervention provided by lay refugee health workers on child malaria treatment-seeking practices among rural refugee mothers of children aged 6 months to 5 years. Refugee health workers were trained to provide health education using local beliefs, terminology, and disease concepts via one-to-one discussions, informal group meetings, and disseminated informational materials. Eight refugee villages were selected; four were assigned to an intervention and four to a control arm. A post-intervention survey of 223 households from the intervention villages and 177 households from control villages revealed a significant difference in positive treatment-seeking behaviors in parents of children who had been exposed to malaria education materials provided by a lay health worker.

**Howard et al. [Guinea]** (19). The objective of this study was to assess the effects of peer-refugee health education on maternal health knowledge, attitudes, behaviors, and maternal health outcomes. Using a refugee self-help model, refugee women mobilized other women who had expertise as nurses, and midwives, as well as other local lay refugee women, as health educators to provide education, make referrals, and distribute contraceptives to women in refugee communities. Local refugees were recruited and trained to interview same-sex respondents. A cross-sectional survey on sexual and reproductive health attitudes related to family planning was administered to 889 participants (445 men; 444 women). Contraceptive use was shown to be markedly higher in areas served by refugee health workers than in the refugees’ under-served countries of origin or host countries (17% vs. 3.9% and 4.1%, respectively).

**Howard et al. [Guinea]** (20). This was a cross-sectional survey that assessed the effects of peer-refugee-led health education related to the danger signs of complications during pregnancy, knowledge of the importance of skilled attendants at birth, the use of family planning and sexual health services, and the use of ante-natal care services among 444 reproductive-age Liberian and Sierra Leonean women in 48 camps across the Forest Region of Guinea. Participants were considered ‘exposed’ to this education if they had previously participated in a peer-refugee facilitated drama group or received information on family planning by a peer-refugee health worker. Although no difference was found in maternal knowledge or attitudes, those exposed to peer refugee-led activities had higher odds of delivery in a health facility than those who were unexposed (OR 2.13, 95% CI 1.21–3.75).

**Lee et al. [Myanmar]** (26). This study used a pre- and post-test design to assess the impact of training and deploying internally displaced villagers to conduct malaria control interventions among IDPs. Staff from the local health department trained internally displaced villagers who lived in the malaria program's target communities and were familiar with other village members. These village health workers were trained in malaria diagnosis, treatment, and vector control. Specifically, they were trained to perform a comprehensive set of malaria interventions, including promoting of the use of long-lasting malaria nets, detecting of early signs and symptoms of malaria, and providing treatment in an active civil conflict area. The intervention demonstrated that internally displaced villagers were able to deliver essential malaria control interventions in areas of active conflict in eastern Burma. Program coverage was expanded from 3,000 to 40,000 internally displaced villagers in 5 years.

**Minden [Thai-Myanmar Border]** (27). This study used a pre- and post-test design to assess the impact of using trained refugee community health workers and traditional birth attendants to provide basic reproductive health services and to diagnose and treat common illnesses among refugees in camps. Qualitative assessment revealed that the trained refugee health workers were able to diagnose and treat common illnesses using drugs, injections, and intravenous infusions according to Medecins Sans Frontieres’ guidelines (28). The trained health workers were able to prevent problems, recognize illness early, and provide treatment before complications escalated to emergencies. They were also able to foresee potential emergencies and were able to stabilize the mothers and/or babies while seeking transportation to a hospital.

**Mullany et al. [Myanmar]** (24). This study used a pre- and post-intervention design to assess the feasibility of a network of community-based providers to deliver maternal health interventions in the complex emergency setting of eastern Burma. In target communities, lay health workers and traditional birth attendants were trained in basic emergency obstetric care, blood transfusion, antenatal care, and family planning. A post-intervention survey was conducted to assess the effects of the intervention on accessing ante- and post-natal care, skilled attendants at births, and family planning services. Results showed that, following intervention, use of insecticide-treated mosquito nets increased, as did use of modern contraceptives. Births attended by those trained to deliver elements of emergency care increased about 10-fold.

**Rijken et al. [Thai-Myanmar Border]** (25). This study used a pre- and post-test design to assess the effects of a 3-month practical and theoretical training for lay refugee health workers aimed at equipping them with skills in conducting obstetric ultrasound imaging based on World Health Organization guidelines and British Medical Ultrasound Society recommendations. Post-test assessments measured intra-observer and inter-observer agreement of fetal biometry in 349 pregnant women measured by trained displaced health workers compared to those conducted by an expatriate physician. Measurements by refugee health workers showed high levels of agreement with those of the physician, demonstrating that locally trained health workers from refugee camps could adequately conduct obstetric ultrasound imaging.

**Tanaka et al. [Tanzania]** (22). This was a cross-sectional study in the Lugufu refugee camp in Tanzania, which hosted refugees from Burundi, Rwanda, and the Democratic Republic of Congo (DRC). At the time of the study, Lugufu Camp was hosting an estimated 50,400 Congolese refugees, with an average of 1,000 people arriving each month as a result of the continuing conflict in the DRC (22). The camp had a health information team (HIT) comprised of Congolese refugees (one HIT member/1,000 population) who provided health services under the supervision of health staff of the Tanzanian Red Cross Society (TRCS) (22). The majority of the sampled community members and TRCS health staff affirmed the positive contribution of HIT to refugee health (89.2 and 100%, respectively). Seventy-nine percent of the sampled refugee community members reported that they learned about illness prevention from the HIT. Also, HIT was the education method most highly rated by the refugee community, both for learning how to prevent illnesses (56.3%) and for learning how to treat mild diarrhea (50.0%). The role played by the HIT as a liaison between the refugee community and health services was recognized by 85.2% of the refugees surveyed; refugee community members who did not know a HIT member demonstrated less positive health seeking behaviors than those who knew one or more HIT members (22).

**Woodward et al. [Guinea]** (21). This was a cross-sectional study designed to assess the association between exposure to refugee peer education and improved HIV knowledge, attitudes, or practice outcomes among refugees in Guinea. Data were collected from 889 reproductive-age men and women in 23 camps in the Forest Region of Guinea, and exposure to peer refugee led education was analyzed and compared with HIV outcomes using logistic regression odds ratios. The results of these analyses showed that exposure to peer refugee health education was associated with awareness of HIV/AIDS and reduced misconceptions about the disease. Overall, participants who were exposed to peer refugee education had more than twice the odds of reporting having made HIV-avoidant behavioral changes than those who were unexposed (72% versus 58%; adjusted OR 2.49, 95% CI 1.52–4.08) (21).

## Critical appraisal of data on impact of use of lay refugee health workers

As shown in Table 1, all of the included studies demonstrated some positive impact on population health outcomes as a result of training and deployment of lay refugee/IDP health workers in camps. The reported effects included improvements in knowledge, attitudes, and practices related to various sexual, reproductive and other maternal and child health issues. Specific examples included increased knowledge about the symptoms and prevention of STIs, improved treatment-seeking and protective behavior adopted by those experiencing STI symptoms, and uptake of family planning services. Other reported changes included improvements in child fever and malaria treatment-seeking behaviors, and improved access to reproductive health information. One study, which assessed the effect of peer refugee health education on maternal health knowledge, attitudes, behaviors and maternal health outcomes (19), did not demonstrate marked reduction in unintended pregnancies among refugee women. For the purpose of this review, study quality assessment revealed that all included studies were of poor quality. For example, in five of the included studies (12, 18–21) , the analysis did not separate impact of interventions delivered by refugee nurses and midwives from those delivered by trained refugee women who served as lay health workers. Cropley (23) used a post-test only design. The lack of comparability of intervention and control communities at baseline raises concerns about internal validity (29), i.e. the approximate truth of inferences regarding causal relationships. Although the study by Lee et al. (26) demonstrated the ability of trained displaced villages to contribute to significant expansion of malaria treatment and prevention services, it did not assess the quality or outcomes of services provided by the trained lay refugee villagers. Minden (27), Mullany et al. (24), and Rijken et al. (25) used the before-and-after design in assessing the effectiveness of trained lay refugee community health workers and traditional birth attendants in providing basic reproductive health services, and in diagnosing and treating common illnesses. Before-and-after design is relatively cheap to implement and useful in addressing potential ethical concerns that may be associated with randomized studies or prospective cohort designs. However, the lack of a comparison group limits the degree to which observed health outcomes can be attributed to services provided by lay refugee workers. Outcome measures assessed by all studies (12, 18–27) were based on self-reports, which are known to be subject to the effects of social desirability (30). More importantly, none of the studies assessed the relationship between the degree of exposure to interventions provided by lay refugee workers and health outcomes.

## Discussion

Globally, a shortage of human resources for the health sector is widely acknowledged as a key barrier against the provision of essential health services (31). The burden of health workforce shortage is more acute in low-income countries, especially those experiencing conflicts and emergencies, when the few available health workers are forced to flee, health infrastructures are destroyed, and resources for the health sector are diverted to other uses. Available evidence shows that community health workers have the potential to be part of the solution to the human resource crisis that is affecting many countries (32, 33), and scaling up training and deployment of community health workers is one of the strategies identified in the Kampala Declaration and the Agenda for Global Action (34). A systematic review conducted by the Global Health Workforce Alliance and the United States Agency for International Development (USAID) to elucidate the effectiveness of community health workers concluded that adequate training, integration, and supervision of community health workers has the potential to contribute to an equitable and cost-effective scale-up of service coverage, while leading to tangible improvements in health outcomes (28).

In light of evidence regarding the effectiveness of lay workers in population health improvement, there is a need to re-evaluate how camps for refugees and IDPs are organized and the way services are provided for women and children who reside in camps. With a five-fold increase in emergencies caused by natural disasters and/or conflicts over the past decade, it has become increasingly difficult for international relief agencies to keep pace (35). Camps are resource-limited settings. As such, available resources and services become quickly depleted with time. There is a need to train and effectively deploy lay refugees and IDPs in order to sustain access to health basic health services. Lay-refugee and internally displaced women who reside in camps have life experiences that are similar to those of other women in the camps. They also have valuable health-related cultural knowledge that expatriates may not have (17, 31). Thus, there is the perception that, if trained and adequately supported, they can be well-positioned to provide culturally appropriate support that is better targeted to the needs of women and children in the camps. Similar to the village health-worker and promotora approaches that have been shown to be beneficial in helping to meet the healthcare needs of women and children in resource-poor settings globally (36), lay refugees and IDPs who reside in camps could potentially help ease reliance on foreign health professionals, improve access and coverage, and empower women to enhance their health and the health of their children and families. Whereas anecdotal evidence suggests that training and deployment of lay refugees and IDPs have public health benefits, no reviews have sought to assess and critically appraise the evidence base of this intervention to facilitate policy recommendations. Therefore, in order to address this gap in knowledge, we conducted a systematic review and critical appraisal of available data.

An exhaustive search of the literature yielded only 10 studies that attempted to assess the role of lay refugees and IDPs in reproductive health service provision in camps. All of the included studies reported some positive impact association with training and deployment of lay refugee/IDP health workers in camps. The reported effects ranged from improvements in knowledge, attitudes, and health seeking behavior; an increased efficacy to treat mild diarrhea; a capacity to conduct obstetric ultrasound imaging; an ability to diagnose and treat minor conditions (including malaria and STIs); to an increased uptake of family planning services. While these findings are important, the current body of evidence is weak, and there remains a paucity of high quality evaluation studies of the impact of using trained lay refugees and IDPs to provide health services to camp dwellers.

## Limitations

As noted earlier, this review included studies that used a wide variety of designs (cross-sectional, post-test only, and pre- and post-test) that have significant inherent limitations, especially regarding internal validity. For example, cross-sectional studies measure exposure and health outcomes simultaneously. Thus, it is difficult to determine the direction of the observed associations. Post-test only studies lack baseline comparability of intervention and control communities; before-and-after studies lack appropriate comparison groups. Although many international health agencies are involved in emergency response activities globally, none was able to provide information on evaluated studies of interventions that involved the use of refugees and/or IDPs to provide maternal and child health services in camps. This raises concerns about the value placed on evidence-based practice in international emergency response activities. However, it should be understood that camps are resource-limited settings. Under emergency situations, where the primary health objective is to ensure safety and access to basic services for refugees and IDPs, it may be difficult to commit limited resources to planning and implementing rigorous evaluation studies. It may also be difficult to employ such rigorous evaluation designs as the randomized controlled trial (RCT) due to ethical reasons. Thus, notwithstanding these challenges, it is important to note that time, money, and efforts expended on activities of questionable impact have opportunity costs. To the extent that is reasonably practicable, interventions that train and deploy lay refugees and IDPs as healthcare workers should be rigorously evaluated in order justify resource.

## Implications for research and practice

To further elucidate the strength of the association between training and deployment of lay refugee/IDP health workers and the quality of basic health services in camps, there is a need for high quality follow-up studies conducted in different geographical regions of the world and among individuals of diverse racial/ethnic cultural backgrounds. Currently, available evidence reflects an urgent need for adopting a more evidence-based practice approach. While it is true that the most important objective when organizing such training interventions as described above, is to promote population health, efforts should also be made where possible to strengthen the mechanisms for collecting the best evidence to advocate for more training programs and to facilitate the replication of best practices in other settings. Studies that assess the quality of services provided by lay refugee/IDP health workers in camps are needed, as are studies that demonstrate the effects of such hard outcome measures as reduced morbidity and mortality or positive measures of health and wellness, rather than simply changes in knowledge and attitudes.

## Conclusion

While available evidence suggests a positive impact of training and deployment of lay refugee/IDP health workers to provide basic maternal health services in camps, the body of evidence is weak and calls for a re-examination of current practices. It is important that every intervention includes a strong evaluation component to better elucidate the extent to which changes in outcomes can be attributed to interventions provided by lay refugee/IDP health workers in camps.
